# Variation in RNA expression and genomic DNA content acquired during cell culture

**DOI:** 10.1038/sj.bjc.6601405

**Published:** 2004-01-20

**Authors:** L R Hiorns, T D Bradshaw, L A Skelton, Q Yu, L R Kelland, B Leyland-Jones

**Affiliations:** 1 Department of Experimental Haematology, St Bartholomew's and the Royal London School of Medicine, Turner Street, London E1 2AD, UK; 2 CRC Experimental Cancer Chemotherapy Research Group, University of Nottingham, Nottingham NG7 2RD, UK; 3 CRC Centre for Cancer Therapeutics, Institute of Cancer Research, Sutton, Surrey, UK; 4 Department of Oncology, McGill University, 546 Pine Avenue West, Montreal, PQ, Canada H2W 1S6

**Keywords:** CGH, expression arrays, cell culture, breast cancer, ovarian cancer

## Abstract

Specific chromosomal abnormalities are increasingly recognised to be associated with particular tumour subtypes. These cytogenetic abnormalities define the sites of specific genes, the alteration of which is implicated in the neoplastic process. We used comparative genomic hybridisation (CGH) to examine DNA from different breast and ovarian cancer cell lines for variations in DNA sequence copy number compared with the same normal control. We also compared different sources of the MCF7 breast line by both CGH and cDNA expression arrays. Some of the differences between the subcultures were extensive and involved large regions of the chromosome. Differences between the four subcultures were observed for gains of 2q, 5p, 5q, 6q, 7p, 7q, 9q, 10p, 11q, 13q, 14q, 16q, 18p and 20p, and losses of 4q, 5p, 5q, 6q, 7q, 8p, 11p, 11q, 12q, 13q, 15q, 19p, 19q, 20p, 21q, 22q and Xp. However, few variations were found between two subcultures examined, 5 months apart, from the same initial source. The RNA arrays also demonstrated considerable variation between the three different subcultures, with only 43% of genes expressed at the same levels in all three. Moreover, the patterns of the expressed genes did not always reflect our observed CGH aberrations. These results demonstrate extensive genomic instability and variation in RNA expression during subculture and provide supportive data for evidence that cell lines do evolve in culture, thereby weakening the direct relevance of such cultures as models of human cancer. This work also reinforces the concern that comparisons of published analyses of cultures of the same name may be dangerous.

Long-term culture *in vitro* has represented the most commonly used experimental model of cancer for the past 40 years (Goldin *et al*, 1979, pp. 165–245; Boyd, 1989, pp. 1–12; Monks *et al*, 1991). Even though the focus of the NCI screen has changed in recent years to become more tissue specific and molecularly targeted, heavy reliance is still placed on well-characterised long-term cell culture lines. This is of concern since phenotypic diversity has been demonstrated between established cell lines and the primary tumour from which they were cultured (Band *et al*, 1990; Lukashov and Goudsmit, 1995; Sen *et al*, 1995). Moreover, differences in biological properties have been demonstrated between sublines of both haematopoietic and breast cell lines (Osborne *et al*, 1987; Band *et al*, 1990; Crepin *et al*, 1990; Lukashov and Goudsmit, 1995). However, long-term subcultures have not been examined for variation in expression profiling and genomic (chromosomal) abnormalities.

In this study, we report considerable variation between different cultures of the MCF7 cell line and lesser variation between different cultures of three ovarian cell lines. The extent of differences was more pronounced for a particular cell line interlaboratory than within the same laboratory at different time points. These results provide supportive data for evidence that cell lines do evolve in culture, thereby weakening the direct relevance of such cultures as models of human cancer. This work also reinforces the concern that comparisons of published analyses of cultures of the same name may be dangerous.

In order to identify new drug resistance genes, we have applied the technique of comparative genomic hybridisation (CGH) (Kallioniemi *et al*, 1992) to screen for chromosomal abnormalities specific to the acquisition of resistance in long-term culture cell lines (Leyland-Jones *et al*, 1999). We have demonstrated the validity of this approach by identifying high-level amplifications of genes known to be associated with specific resistance mechanisms (Hiorns *et al*, 1999). CGH ratios were determined for a series of breast and ovarian carcinoma cell lines. We noted considerable variation in the CGH patterns between two cultures of the same cell line obtained from different laboratories. This observation prompted us to investigate further cell lines. For four of the cell lines (one breast and three ovarian), we made a comparison of different sources of the tumour cell line (against the same normal control). For one breast (MCF7) and one ovarian (CH1) cell line, we made a comparison of the aberrations present in cultures from the same laboratory 5 months apart. For one cell line (MCF7), we obtained cultures that had been independently maintained in different geographical locations over a number of generations. In order to compare the expression levels of certain genes with their genomic representation, we used cDNA arrays (Botwell, 1999) to examine different patterns of RNA expression between these different cultures.

Among the methods available for comprehensive analysis of RNA expression, cDNA microarrays were chosen for their ability to detect static information on specific gene expression and dynamic information on the levels of gene expression in a single experiment. From the commercially available microarrays, we chose the Atlas™ cancer arrays (Clontech, Palo Alto, USA) since the cDNAs on the filters are known genes preselected for their involvement in cancer and grouped according to their involvement in specific processes. The sensitivity of this filter-based, ^32^P-probe-labelled methodology is known to be limited to high and medium abundance genes.

## MATERIALS AND METHODS

### Cell lines

These cell lines were deliberately chosen to represent realistic diversity in the scientific community. In particular, for the MCF7, the McGill lines came from an NIH source; the two differently timed aliquots were carefully controlled in the senior author's laboratory. The Nottingham and ICR cell lines were both obtained from ATCC and were obviously grown in different laboratories, but under conditions suitable for collaboration.

A series of cell lines previously established from breast and ovarian carcinomas were passaged independently for a number of years in long-term culture. Cell lines MCF7(b), A2780(a), 41M(a) and CH1(a) were obtained from the laboratory of LRK; cell lines OVCAR-3, A2780(b), 41M(b) and CH1(b and c) from the laboratory of LAS; cell lines MCF7 (c and d) from the laboratory of BL-J; and cell line MCF7(a) from the laboratory of TDB. Two aliquots of the MCF7 cell line (c and d) and two of the CH1 cell line (b and c) were sampled 5 months apart, having been kept in continuous passage during the intervening period.

41 M and A2780 are ovarian carcinoma cell lines established from previously untreated patients. OVCAR-3 was established from the malignant ascites of a patient with progressive ovarian adenocarcinoma. CH1 was established from an ovarian carcinoma patient previously treated with, and resistant to, cisplatin and carboplatin. MCF7 is a breast cancer cell line established from a previously untreated patient. All cell lines were aneuploid. All cell lines were maintained as monolayers in Dulbecco's minimum essential medium supplemented with 10% foetal calf serum, 50 *μ*g ml^−1^ gentamicin, 0.5 *μ*g ml^−1^ hydrocortisone and 2 mM L-glutamine, in a 5% CO_2_ atmosphere. For the RNA expression studies, all three MCF7 cell lines were plated at the same densities, appeared to grow at equal rates and were sampled at the same time points.

### CGH

DNA was extracted from harvested cells by the conventional phenol/chloroform technique. DNA (1 *μ*g) from each cell line was labelled for 20 h with Biotin-High-Prime (Boehringer Mannheim, Lewes, UK) using their supplied protocol. ‘Normal’ control DNA (1 *μ*g) was labelled with Digoxigenin-High-Prime (Boehringer). Unincorporated nucleotides were removed using Nick columns (Pharmacia, St Albans, UK). Equal amounts (1 *μ*g) of each DNA were combined together with a 50-fold excess of COT1 DNA (Gibco/BRL, Paisley, UK), ethanol precipitated and resuspended in 10 *μ*l Hybrisol VIII (Oncor, Gaithersburg, MD, USA). This probe mixture was then denatured at 75°C for 8 min, chilled on ice and warmed to 37°C, followed by hybridisation to slides containing ‘normal male’ lymphocyte metaphases (Vysis, Downers Grove, IL, USA). Hybridisation and washing conditions were described previously as (Hiorns, in press).

Slides were examined using an Axioskop microscope (Carl Zeiss, Welwyn Garden City, UK) equipped with appropriate filters. Images were collected using a CCD camera (Photometrics, Tucson, AZ, USA) and Quips™ software (Vysis). In order to control against small changes in hybridisation efficiency, at least 10 metaphases were captured for each hybridisation; the chromosomes were karyotyped and the axis defined based on the DAPI banding pattern. The DAPI image as used as a mask for the red and green images to exclude background fluorescence. The total image intensity for the masked red and green images was then independently normalised to accommodate differences in the image capture, so that the average red : green ratio for each cell was 1.0. The red : green ratios for cross-sections of each chromosome were measured perpendicular to the axis. The ratio profiles of 10 metaphases were averaged and represented graphically. Chromosomal imbalances affecting more than 30% of the cell population were identified when the ratio was greater than 1.15–1.20 for gains and less than 0.85–0.80 for losses (all imbalances were within 99% confidence limits). The heterochromatic regions consisting of clustered repetitive sequences were excluded from the analysis as were the telomeres of all chromosomes.

### Expression arrays

Total RNA was extracted from cell lines in exponential growth phase, using the Atlas™ Pure RNA Isolation Kit (Clontech, Palo Alto, USA) including all optional purification steps. A measure of 1 *μ*g of each RNA was reverse-transcribed using gene-specific primers and Moloney murine leukaemia virus in the presence of [*α*-^32^P]dATP. The cDNA probes were purified from unincorporated nucleotides using Chroma Spin-200 columns (Clontech, Palo Alto, USA) and hybridised overnight at 68°C to Atlas™ cancer arrays (Clontech, version 1 which analyses 588 human cDNA's, nine housekeeping cDNA's and negative controls). A series of high-stringency washes were performed, using the protocol supplied. The hybridisation pattern was detected using a phosphorimager. Signals greater than twice the background were considered positive. The ratio of signal intensity for the expressed genes was compared to that of the housekeeping genes using AtlasImage™ software (Clonetech). Comparisons between the cell lines were carried out on a paired basis using the same software. Differences between expression levels were considered significant according to the accepted criteria of (i) a ratio difference of more than two-fold, or (ii) an intensity difference of more than the two backgrounds combined.

## RESULTS

### Breast cancer cell line CGH results: overall agreement with literature

The genomic imbalances identified in the MCF7 cell line are in broad agreement with the frequently observed changes in the literature, including both those reported for primary human breast carcinomas (Nishizaki *et al*, 1997; Tirkkonen *et al*, 1998) and cell lines (Kallioniemi *et al*, 1994; Jones *et al*, 2000). The most frequently observed changes in our four different MCF7 subcultures included consistent gains of 1q, 3p, 3q, 8q, 12q, 15q, 17q and 20q together with persistent losses of 9p, 17p, 18q and X. In particular, we observed the gains 3p14 and 14q reported by Kallioniemi *et al* (1994) but not observed by Jones *et al* (2000). However, the gain of 14q was only observed in the cell lines from one of our three laboratories (subcultures c and d).

### Striking differences between four MCF7 subcultures

**Figure 1:**
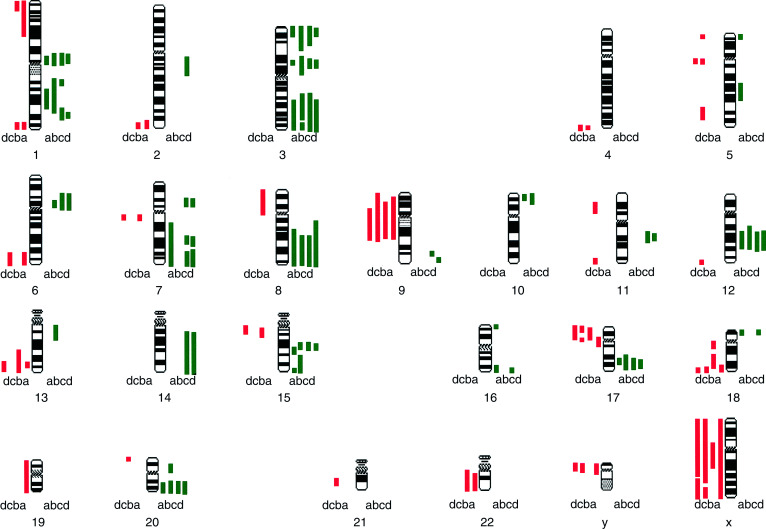
Idiogram of the human karyotype showing regions of amplification (green) and deletion (red) in DNA from the MCF7 cell line from different sources: (a) University of Nottingham; (b) Institute of Cancer Research (Sutton); (c) McGill University (Montreal) April 1996; and (d) McGill University (Montreal) August 1996.

We note surprising variation in the chromosomal aberration patterns between the four different MCF7 subcultures (illustrated in Figure 1

). The following differences between the four subcultures were observed: gains of 2q, 5p, 5q, 6q, 7p, 7q, 9q, 10p, 11q, 13q, 14q, 16q, 18p and 20p; and losses of 4q, 5p, 5q, 6q, 7q, 8p, 11p, 11q, 12q, 13q, 15q, 19p, 19q, 20p, 21q, 22q and X. Some of the differences between the subcultures were extensive involving large regions of the chromosome. Many of the changes between our cell lines are similar to those observed by Jones *et al* (2000). It is reassuring to note fewer variations between the two subcultures from the same laboratory (c and d) which were examined over a period of 5 months in continuous passage. In all, 15 differences were noted between subcultures (c) and (d) in contrast to 22 differences between (a) and (b), 30 differences between (a) and (c), and 34 differences between (b) and (c).

### Fewer CGH differences between ovarian cell line subcultures

The genomic aberrations identified by CGH in the ovarian cell lines studied (A2780, CH1 and 41M) are listed in Table 1

**Table 1:** Amplifications (green) and deletions (red) of genomic sequences identified in ovarian tumours and the ovarian tumour-derived cell lines: Ovcar, A2780, 41M and CH1

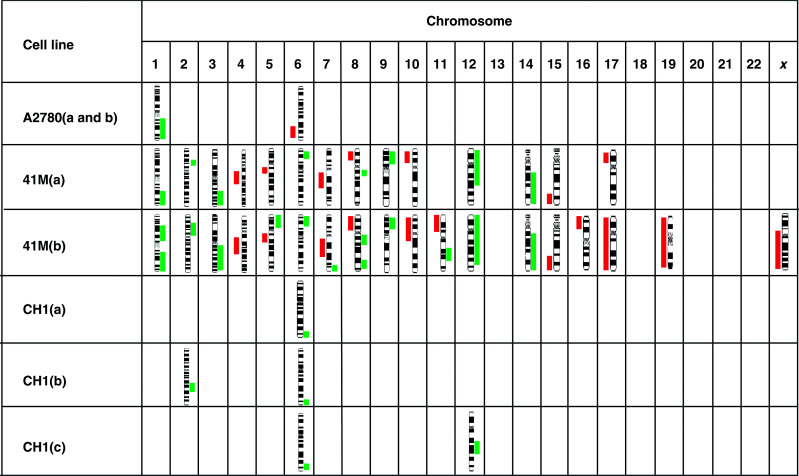

. The ovarian cancer cell lines showed a wide variation in the number and location of genomic imbalances. In contrast to the MCF7 breast line, each ovarian cancer cell line showed a much smaller degree of variation both between different laboratories and with time.

### Similarities between MCF7 subculture expression patterns

**Figure 2:**
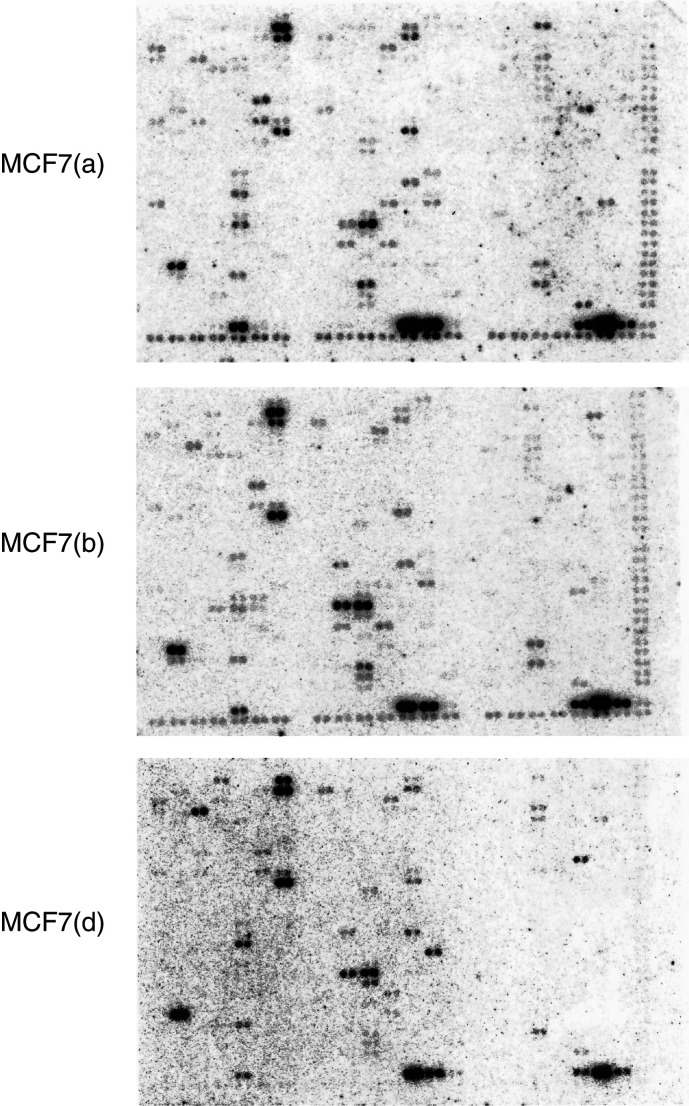
Clonetech ‘Cancer Array’ filters hybridised with cDNA from MCF7 sublines from different sources: (a) University of Nottingham; (b) Institute of Cancer Research (Sutton); and (d) McGill University (Montreal). Each hybridisation was to a new replicate filter; (none were reprobed).

Similar patterns of RNA expression were observed for all three of the MCF7 subcultures (a, b and d) both with respect to specific genes and the levels of gene expression (see Figure 2

). In all, 116 genes were expressed at levels greater than 0.01% in one or more of the cultures; this number is similar to that reported by Sehgal *et al* (1998) in GMTT glioblastoma cells but less than that reported by Xie *et al* (1998) in HL-60 leukaemia cells. A total of 52 genes showed the same relative levels of expression in all three cultures.

### Sixty-four genes demonstrated variability in levels of expression between the different subcultures

The chromosomal location and relative expression levels for these genes are listed in Table 2

**Table 2:** Comparative expression levels of the 45% of genes differentially expressed between at least two of the cell lines

**Chromosome location**	**Gene name**	**MCF7 (a)**^a^	**MCF7 (b)**^a^	**MCF7 (d)**^a^
1p13.21	rhoC	3.87	6.48	3.69
1p13.3	Glutathione-*S*-transferase (GST)	7.40	3.57	3.47
2p10–12	rhoB	15.29	10.63	3.61
2q33–34	IGFBP2	—	6.43	—
3p21.3	rhoA H12	14.92	7.02	7.23
3p21.3	Laminin receptor	18.21	15.00	9.11
3p21.3	Semaphorin	—	3.54	—
4q12	PDGF-associated protein	7.49	—	—
5q12	CyclinB1 (CCNB1)	3.61	—	—
5q31.3	Early growth response protein (EGR1)	8.21	7.48	—
5q31	Interleukin-13 precursor	3.70	3.49	—
5q31	Alpha 1 catenin	3.69	—	—
6p21.2	CDKN1,WAF1	6.62	6.45	9.06
8p12	T-plasminogen activator	5.98	4.11	—
9p12	Bcl-2 binding athanogene-1 (BAG-1)	3.95	—	—
9q22	Ninjurin-1	8.20	5.58	5.45
9q34.3	NOTCH1	3.82	—	11.22
10q24	Cytohesin-1	4.26	4.02	2.63
11p13	CD59	5.24	—	—
11p15.4–15.5	rhoG	3.57	—	3.42
11q13	Fau	12.61	—	16.58
12p13	CD9	11.94	5.48	—
12p13	CD27BP /SIVA	13.97	4.93	8.81
12q13	Cytokeratin 8	17.17	15.00	9.10
12q13	Cytokeratin 18	18.21	15.00	9.10
12q14	CDK4	6.32	3.00	3.75
14q24.3	c-fos	3.35	3.46	—
16p11.2	MAP kinase 1	3.95	—	2.28
16p13.3	Netrin-2	—	6.97	—
16p13.3	Nm23-H4	3.70	5.53	8.03
17q21–22	Cytokeratin 10	11.06	3.42	4.46
17q21–22	Cytokeratin 19	18.17	13.85	9.11
17q21	Desmoplakin III	6.57	6.39	13.33
17q21.3	NDP kinase A (NM23-H1)	4.69	4.93	3.05
17q21.3	NDP kinase B (NM23-H2)	18.21	15.00	11.59
17q21.32	Integrin beta 3	—	3.79	—
17q24–25	EGFRBP-GRB2	3.49	—	—
17q25.3	Rho GDP dissociation inhibitor 1	8.53	5.99	10.10
19p13.2	UV excision repair protein RAD23A	5.65	—	14.71
19p13.3	CDC34	—	3.06	4.78
19q13	Apoptosis regulator bax	4.29	4.15	5.14
19	Macrophage migration inhibitory factor (MIF)	12.31	3.93	11.62
20p13	CDC25B	4.29	—	2.56
21q22.1	Cytosolic superoxide dismutase (SOD1)	13.73	3.21	7.53
22q11	DVL1	3.90	—	—
Xp11.23	PCTK1	—	—	2.84
Xp11.23–3	EPA/TIMP-1	—	6.95	4.26
Xq25	Hepatoma-derived growth factor (HDGF)	10.00	6.93	19.53
	CD147 antigen	7.13	14.41	13.39
	Zyxin-related protein ZRP-1	10.12	—	8.08
	Cytokeratin 7	5.30	5.60	5.38
	Cytokeratin 16	5.24	4.47	—
	rhoHP1	5.84	7.10	—
	P21-rac1, ras-like protein TC25	5.85	—	—
	PLK1	4.80	—	—
	PDCD2	4.14	—	—
	CASP2	3.22	—	—
	FAST kinase	3.94	—	3.66
	CDK5	4.02	—	2.50
	c-myc-binding protein MM-1	13.06	6.85	4.56
	Cytokeratin 5	—	—	2.80
	PIG12	—	—	4.12
	Integrin-beta 8 precursor	—	—	2.30
	IGFBP5	—	—	3.49

a Ratio of signal intensity to background. MCF7 sources: (a) University of Nottingham, (b) Institute of Cancer Research (Sutton), and (d) McGill University (Montreal).

. All 64 genes either showed a ratio difference greater than two-fold or an intensity difference between two subcultures greater than the two background intensities combined (with the vast majority of differences greater than six-fold).

Differences in gene expression (for example, ZRP-1, fau, RAD23A) between some of the subcultures were profound. Moreover, large differences were observed in expression levels of key genes involved in multiple cellular processes – signal transduction, cytoskeletal maintenance, transport, adhesion, oncogenesis, apoptosis, etc.

### Characteristics of highly expressed genes common to all subcultures

The majority of genes expressed at high levels in MCF7 cultures were either intermediate filament markers (1) or genes involved in their anchorage (2); for example:

1. Cytokeratin 8, cytokeratin 18 and WAF-1, which have previously been shown to be differentially expressed in MCF7 cells (in contrast to MDA-MB 231) (Kuang *et al*, 1998).
2. All three cultures had low levels of expression of rhoC and rhoG, and all but one had higher levels of expression of rhoA and rhoB. Absolute levels of expression varied widely between the three different cultures.

The other notable observation involved the two heterodimeric subunits (H1 and H2) of the metastasis inhibition factor NM23. NM23-H2 was consistently expressed at high levels, and NM23H1 was consistently expressed at low levels in all subcultures. Low levels of NM23 correlate with high metastatic potential (Steeg *et al*, 1988); they are believed to complex with G-proteins and affect developmental pathways.

## DISCUSSION

The viability of using long-term cell lines as models of human tumours has been questioned in a number of reviews (Corbett *et al*, 1987; Orth *et al*, 1994; Sen *et al*, 1995). It is well known that cells are subject to selection both during initial culture and subsequent long-term propagation. Phenotypic diversity has been demonstrated between established cell lines and the primary tumour from which they were cultured (Band *et al*, 1990; Lukashov and Goudsmit, 1995; Sen *et al*, 1995). Differences in biological properties (morphology, phenotype, karyotype, growth rate, cloning efficiency and tumorigenicity) have been demonstrated between sublines of haematopoietic cells (Band *et al*, 1990; Lukashov and Goudsmit, 1995) and for the breast cell lines MDA-MB 231 (Crepin *et al*, 1990) and MCF7 (Osborne *et al*, 1987). Murine tumours derived from long-term cell culture passage are frequently less invasive, far less metastatic and less aggressive than the same tumour maintained by animal passage only (Corbett *et al*, 1987). The drug sensitivities for long-term passaged tumours *in vitro* can, and do, change frequently (Corbett *et al*, 1995; Valeriote *et al*, 1996). In contrast, the sensitivity pattern for a tumour passaged *in vivo* to a given set of agents remains relatively stable for many years and dozens of transplant generations. Microsatellite instability has been demonstrated in ovarian cell lines (Orth *et al*, 1994) and is further increased in resistant sublines (Anthoney *et al*, 1996). Recently, mutations in hMSH2-deficient tumour cell lines have been shown to accumulate in a time-dependent manner in the absence of growth (Richards *et al*, 1997). This situation is perhaps more representative of the microenvironment of a tumour than the rapid growth that occurs in cell culture.

Our CGH results for the four MCF7 subcultures show moderate variations in two subcultures of the same parental line within the same laboratory but striking differences between subcultures from three different laboratories. Whereas Jones *et al* (2000) have already demonstrated extensive variation among different MCF7 cell stocks, this is the first report that:

- compares and contrasts MCF7 lines from different laboratories;
- examines the same subculture taken at different time intervals; and
- most importantly, compares the CGH analytical work with expression array results.

**Figure 3:**
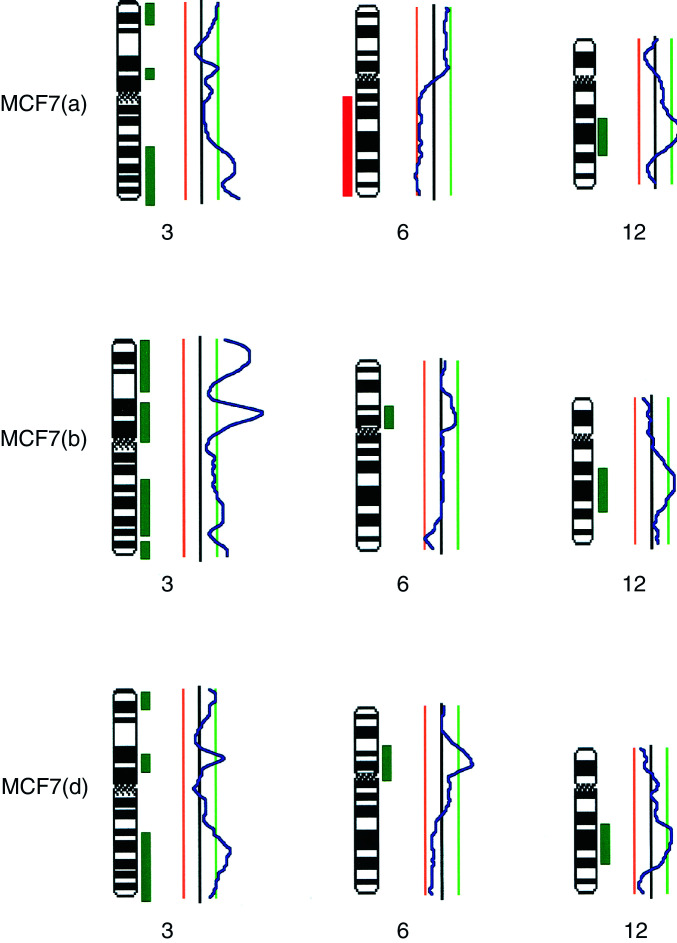
CGH ratio profiles and accompanying idiograms for selected chromosomes for the three MCF7 cell lines: (a) University of Nottingham; (b) Institute of Cancer Research (Sutton); and (d) McGill University (Montreal). The ratio profiles are shown together with the threshold lines for amplification in green on the right and deletion in red on the left. The areas which exceed the threshold are also illustrated in green or red alongside the idiogram. The heterochromatic regions (which are excluded from the analysis) are shown hatched on the idiogram.

Moreover, our cDNA array results showed that the majority of expressed genes were related to intermediate filament markers and cell–cell interaction. Both CGH and expression results demonstrated a surprising level of divergence between subcultures from the different laboratories. Comparison between genomic copy number (as identified by CGH) and gene expression (as measured by arrays) is complicated by the fact that CGH does not resolve the fine structure of genome copy number changes. Thus, some lack of correlation may occur because narrow amplicons are missed in CGH or narrow deletions are missed, if part of a larger amplified region (Figure 3

).

Since all three subcultures were grown in the same media at the same time, harvested in exponential phase and RNA extracted simultaneously, the differences that we observed in gene expression reflect real differences between the subcultures. Substantial variations were observed at both the genomic and RNA expression levels. Each subculture may have been subjected to different selection pressures or have reacted differently to the same selection pressures. Furthermore, overexpression can and does occur in the absence of DNA amplification and *vice versa*.

The combination of CGH and expression arrays may be useful in differentiating between genes that are transitorily expressed at high levels and those for which there is a long-term requirement for high-level expression. We agree with Pollack *et al* (1999) that genes which are both amplified and highly expressed may be more likely candidates for control of tumour initiation or progression.

These results not only question the utility of long-term culture cell lines as a basis for genomic analysis and the identification of signalling pathways, but also provide supportive data for evidence that cell lines do evolve in culture, thereby weakening the direct relevance of such cultures as models of human cancer. This work also reinforces the concern that comparisons of published analyses of cultures of the same name may be dangerous.
